# Anesthetic Challenges in a Pediatric Patient With Chronic Kidney Disease Complicated by Dilated Cardiomyopathy Undergoing Non-cardiac Surgery

**DOI:** 10.7759/cureus.70477

**Published:** 2024-09-29

**Authors:** Shilpa Kore, Vipul Sharma, Ishan Garud

**Affiliations:** 1 Anesthesiology, Dr. D. Y. Patil Medical College, Hospital and Research Centre, Dr. D. Y. Patil Vidyapeeth (Deemed to be University), Pune, IND

**Keywords:** anesthesia management, chronic kidney disease (ckd), dilated cardiomyopathy, end-stage renal disease (esrd), pediatric anesthesia, unilateral renal agenesis

## Abstract

Dilated cardiomyopathy (DCM), a primary myocardial disorder, manifests through the dilation of one or both ventricles, coupled with systolic and valvular dysfunction. Renal agenesis is a congenital condition characterized by the absence of one or both kidneys at birth. Unilateral renal agenesis, wherein one kidney is absent, can subtly evade detection due to the impressive adaptability of the remaining kidney, often preserving typical functionality. Nevertheless, when compounded with chronic kidney disease (CKD), the repercussions of renal agenesis become notably more pronounced. CKD and DCM represent two significant and interrelated clinical challenges, particularly in pediatrics. This case report examines the anesthesia management of a 10-year-old female with CKD and right renal agenesis complicated by DCM undergoing bilateral hemi-epiphysiodesis for genu valgum correction. It emphasizes the crucial role of a multidisciplinary approach in achieving favorable outcomes in patients undergoing non-cardiac surgery.

## Introduction

Chronic kidney disease (CKD) and dilated cardiomyopathy (DCM) present substantial and interrelated challenges, particularly in the pediatric population. CKD, characterized by progressive renal impairment, significantly affects cardiovascular function, often exacerbating or precipitating cardiac dysfunction. DCM, distinguished by ventricular dilation and systolic and valvular dysfunction, involves complex cardiovascular abnormalities that are further complicated by renal impairment. In pediatric patients, the challenges are magnified by developmental and physiological factors. Effective anesthetic management must address the effects of CKD on DCM, requiring judicious selection of anesthetic agents, meticulous fluid management, and rigorous perioperative monitoring. This comprehensive approach is crucial for minimizing the risks associated with these overlapping conditions.

## Case presentation

**Figure 1:**
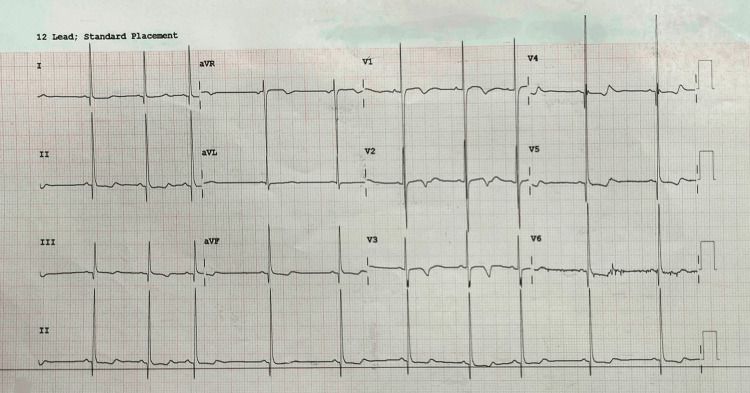
ECG of the patient ECG shows global t-wave inversion, qt prolongation in all leads, and left bundle branch block. ECG: electrocardiogram

A 10-year-old female presented with difficulty walking since birth. She was diagnosed with genu valgum, for which bilateral hemi epiphysiodesis was planned. Antenatally, she was diagnosed with right renal agenesis causing severe maternal oligohydramnios, which led to preterm delivery at 32 weeks of gestation. She became oliguric at one year of age. Ultrasonography revealed hydronephrosis in the left kidney due to pelvicoureteric junction obstruction. Pyeloplasty was done to relieve the obstruction. Post-surgery, the child remained asymptomatic for two years, after which her renal function deteriorated significantly and she was diagnosed with CKD, for which continuous ambulatory peritoneal dialysis (CAPD) was initiated daily. At eight years old and with a body weight of 15 kilograms, she acquired a CAPD catheter-related infection, which progressed to systemic sepsis. On recovery, she was maintained on twice daily CAPD cycles. After this episode, the patient began experiencing symptoms of dyspnea and fatigue. The ECG revealed left bundle branch block, left ventricular hypertrophy (LVH), t inversions, and prolonged q-t interval (Figure 1).

The 2D echocardiography of the patient showed an ejection fraction of 40%, mild central mitral regurgitation, mild pulmonary hypertension, mild left ventricular dysfunction, and DCM. Her cardiac condition was managed by oral digoxin, spironolactone, furosemide, and amlodipine. For her renal health, she was given oral iron, calcium lactobionate, and cholecalciferol. The anesthetic management in this patient involved several unique challenges that required careful consideration. Ensuring hemodynamic stability during induction was critical, with a focus on avoiding tachycardia to prevent further compromise of cardiac function. Precise fluid management was essential to avoid both fluid overload and dehydration; tailored adjustments to the CAPD regimen before surgery were deemed of utmost importance. Intraoperative strategies to prevent and manage arrhythmias and heart failure, given the patient's DCM and reduced ejection fraction, were kept in mind. Advanced hemodynamic monitoring techniques for real-time assessment of cardiovascular status were kept ready inside the operating room. Keeping all the above things in mind and confirming adequate nil per os, the patient was taken inside the operating theater, where she was connected to the standard monitors, and intravenous fluid consisting of 10% dextrose with 0.45% normal saline was initiated via infusion pump. The patient was preoxygenated, followed by premedication with glycopyrrolate (0.004 mg/kg) and midazolam (0.02 mg/kg). Analgesia was provided with fentanyl (2 µg/kg). Induction was achieved using etomidate (0.3 mg/kg), and muscle relaxation was facilitated with cisatracurium (0.15 mg/kg). Following muscle relaxation and laryngoscopy, an endotracheal tube (size 6.0 internal diameter) was successfully placed. The patient was ventilated using volume control mode with sevoflurane at a dial concentration of 1.5% and a mixture of oxygen and air in a ratio of 1:1. Post-induction, an arterial line was inserted into the right radial artery, and a subclavian central venous pressure (CVP) line was placed under ultrasound guidance for potential vasopressor or inotrope administration and CVP monitoring. An epidural catheter was inserted at the L2-L3 level to manage surgical stress and provide postoperative analgesia. The initial top-up included 4 cc of 0.2% ropivacaine combined with 50 µg/kg morphine. Intraoperatively, an infusion of 0.2% ropivacaine with 2 µg/cc fentanyl was administered at 3 ml/hour. Additional cisatracurium was given as needed to maintain neuromuscular blockade. Judicious fluid management to avoid volume imbalances was followed, and the patient’s vital signs remained stable throughout the procedure. Neuromuscular reversal was achieved with neostigmine (0.05 mg/kg) and glycopyrrolate (0.008 mg/kg). The patient was extubated uneventfully and discharged on day 4 with improved ambulatory function.

## Discussion

DCM presents a formidable challenge characterized by left ventricular or biventricular dilation and impaired contractility, devoid of underlying cardiovascular etiologies such as coronary artery disease, hypertension, valvular defects, or congenital heart anomalies [1]. Structural and functional hallmarks include thinning of the left ventricle, ventricular dilation or enlargement, systolic and diastolic dysfunction, global hypokinesia, and cardiomegaly discernible on chest radiography. CVD is present in approximately 50% of individuals with CKD stages 4 and 5 [2]. Cardiovascular-related deaths constitute nearly 40% to 50% of all fatalities in those with advanced CKD (stage 4) and end-stage kidney disease (stage 5), a stark contrast to the 26% mortality rate observed in individuals with normal kidney function [3-4]. Cardiorenal syndrome involves heart-kidney interactions and is classified into five types: cardiac-induced kidney injury (types 1 and 2), kidney-induced heart dysfunction (types 3 and 4), and systemic causes affecting both organs (type 5). Two principal mechanisms contribute to CVD in CKD: the release of hormones, enzymes, and cytokines due to kidney injury [5] and CKD-associated mediators and hemodynamic alterations that exacerbate cardiac damage [6]. Traditional cardiovascular risk factors, such as hypertension and insulin resistance, are highly prevalent in CKD patients and significantly contribute to atherosclerotic vascular disease and CKD progression [7-8]. Increased albuminuria or proteinuria is a potent risk factor for CVD in CKD. Hemodynamic changes in CKD lead vascular smooth muscle cells to switch phenotypes, resulting in accelerated cardiovascular calcifications and increased pulse wave velocity, cardiac afterload, and heart failure [9-10]. These changes induce LVH and decreased coronary perfusion. CKD patients exhibit pathological myocardial fibrosis and cardiac hypertrophy, which are key features of uremic cardiomyopathy. LVH is prevalent in CKD, present in up to 80% of end-stage kidney disease patients, and is an independent survival predictor. Around one-third of dialysis patient mortality is due to sudden cardiac death, mainly caused by atrial arrhythmias. Atrial arrhythmias affect one in three dialysis patients, with a mean of 88.8 arrhythmia episodes per person-year and atrial fibrillation at 37.6 episodes per year [11]. QT interval prolongation and electrical conduction disorders are common, affecting about 50% of hemolysis patients [12]. Hyperkalaemia in 5-10% of end-stage renal disease (ESRD) patients disrupts cardiac conductivity, presenting with peaked T-waves, prolonged PR intervals, and widened QRS complexes. Dialysis procedures can increase arrhythmia risk through electrolyte shifts and volume changes, especially with low-calcium dialysate. Hemodialysis also raises QTc dispersion and interval, and intradialytic hypotension can cause myocardial stunning, increasing cardiovascular event risk and mortality. CAPD aids DCM patients by managing fluid overload, stabilizing blood pressure, and enhancing cardiac function. However, it requires careful oversight due to risks like fluid imbalance, malnutrition, and infection. Anesthetic management for patients with DCM should focus on maintaining adequate diastolic arterial pressure to ensure optimal coronary perfusion, preserving preload, avoiding tachycardia, preventing reductions in myocardial contractility, and ensuring that systemic vascular resistance remains within an appropriate range without becoming elevated [13]. While pharmacotherapy remains pivotal, utilizing ionotropic agents like dopamine and dobutamine to transiently enhance cardiac function mandates judicious use, given their propensity to provoke arrhythmias and myocardial irritability. Milrinone stands out in pediatric settings for its favorable impact on myocardial contractility, whereas amiodarone holds promise in managing symptomatic arrhythmias and myocardial refractoriness. In the anesthetic management of pediatric patients with severe cardiomyopathy, early cardiac catheterization is essential for evaluating cardiac output and pulmonary vascular resistance to determine the feasibility of heart transplantation, particularly if there is no improvement in myocardial function. Renal implications add another layer of complexity to perioperative management, demanding meticulous vigilance over fluid and electrolyte dynamics. For patients with ESRD, strict adherence to restricting intravenous fluids to no more than 1 mL/kg during minor procedures under stable conditions is imperative to prevent fluid overload. Hyperkalaemia should be managed judiciously. Adjust anesthetic management to account for altered drug metabolism in CKD; usage of muscle relaxants like atracurium and cisatracurium is preferred, and use of a peripheral nerve stimulator to monitor neuromuscular blockade is advised. Postoperative care should prioritize regional blocks or continuous epidural infusions to help alleviate the pain, thereby promoting early ambulation and recovery.

## Conclusions

Achieving a successful outcome relies on careful preoperative optimization, thorough intraoperative monitoring, and proactive postoperative care. This case demonstrates how personalized perioperative strategies and teamwork among healthcare professionals are crucial for improving outcomes in young patients with complex health issues undergoing surgery.
